# Ensartinib as a neoadjuvant therapy for stage IIIA non-small cell lung cancer patients with EML4-ALK fusion: a case report and literature review

**DOI:** 10.3389/fonc.2025.1474997

**Published:** 2025-02-25

**Authors:** Hao Zhang, Wei Xia, Yifan Zhang, Shihao Bao, Jingtong Zeng, Xianjie Li, Bo Zhang, Hanqing Wang, Song Xu, Zuoqing Song

**Affiliations:** ^1^ Department of Lung Cancer Surgery, Tianjin Medical University General Hospital, Tianjin, China; ^2^ Tianjin Key Laboratory of Lung Cancer Metastasis and Tumor Microenvironment, Lung Cancer, Tianjin, China

**Keywords:** ensartinib, EML4-ALK fusion, neoadjuvant, non-small cell lung cancer, immune microenvironment

## Abstract

Anaplastic lymphoma kinase (ALK) inhibitors have shown efficacy in treating ALK-positive advanced non-small cell lung cancer (NSCLC) patients. However, the effectiveness of ensartinib neoadjuvant therapy remains ambiguous. Herein, we reported that preoperative systemic treatment with the ALK inhibitor ensartinib can be beneficial for treating initially inoperable tumors. In this study, we present a case of a 60-year-old female patient who was diagnosed with stage IIIA (cT2aN2aM0, ninth TNM stage) lower left lung adenocarcinoma harboring an EML4-ALK fusion. After three months of therapy, the neoadjuvant treatment with ensartinib provided a partial response, with significant tumor and lymph node shrinkage. Preoperative ensartinib neoadjuvant therapy for NSCLC is safe and effective. Nevertheless, clinical trials can be conducted in the future to validate our results. Moreover, we performed multiple immunofluorescence staining analyses on samples before and after neoadjuvant therapy, observed and compared the changes in the expression of relevant immune cells (CD8+ T cells, macrophages, PD-1, and PD-L1), and performed a simple analysis.

## 1 Introduction

Lung cancer is one of the most common cancers worldwide and has high morbidity and mortality rates (1). Histologically, lung cancer can be divided into small cell lung cancer (SCLC) and non-small cell lung cancer (NSCLC), of which NSCLC accounts for 85% of all lung malignancies (2). Typically, stage I or II NSCLCs are treated via surgical resection, with adjuvant results based on pathologic treatment. Conversely, adjuvant therapies such as chemotherapy or radiotherapy and surgical intervention are utilized for managing IIIA NSCLC patients.

Soda et al. initially reported the EML4-ALK fusion gene when they amplified a 3926-bp DNA fragment encoding a 1059 amino acid protein, the fusion protein EML4-ALK, in tumor tissue from a lung adenocarcinoma patient (3). In subsequent experiments, the EML4-ALK gene induced cancerous lesions after implantation into normal lung cells, indicating its oncogenic effect (4). About 5% of NSCLC patients show an identified ALK gene alteration,with EML4-ALK rearrangement being the most common pattern (5). EML4-ALK fusion gene positivity occurs in young NSCLC patients with either no or light smoking history (6). Targeted therapies are more effective than chemotherapy in advanced ALK-mutated NSCLC patients. Compared with those treated with chemotherapy, individuals treated with ALK-targeted agents live longer, experience more significant tumor shrinkage, and display an increased period of continued symptom deterioration (7). In recent years, several breakthroughs have been made in targeted therapy technology, resulting in the introduction of various targeted therapeutic agents for EML4-ALK (8). Several ALK tyrosine kinase inhibitors (TKIs), including crizotinib, alectinib, ceritinib, ensartinib, and buxtitinib, have been approved for treating ALK-positive NSCLC patients (9). For lung cancer patients with ALK mutations, the effect of ALK inhibitor-targeted therapy seems to be far better than that of chemotherapy. Compared with that of lorlatinib, the efficacy of targeted therapy in patients with ALK mutations has significantly improved (10, 11). In an analytical study comparing the efficacy of multiple ALK-TKIs in Asian ALK-positive NSCLC patients, ensartinib emerged as an efficient first-line treatment for Asian ALK-positive NSCLC patients (12).

Ensartinib refers to an oral, highly selective, potent ALK-tyrosine kinase inhibitor (TKI). In March 2022, it was approved as a first-line treatment for patients with ALK-positive locally advanced or metastatic NSCLC by the National Medical Products Administration (NMPA). A study by Ma et al. emphasized the efficacy of ensartinib in Chinese patients with advanced ALK NSCLC and demonstrated that ensartinib showed good clinical activity and an acceptable safety profile in Chinese patients with ALK-positive NSCLC through safety, tolerability, pharmacokinetics, efficacy, and possible pharmacodynamic biomarkers which boosted the approval of ensartinib for the treatment of ALK-positive NSCLC patients in China (13). However, very few studies are available on the efficacy of ensartinib neoadjuvant therapy in NSCLC patients who have undergone EML4-ALK fusion. Here, we report a case of a stage IIIA NSCLC patient who experienced significant tumor shrinkage and successful surgery after 3 months of targeted therapy with ensartinib.We also briefly analyzed the changes in the immune microenvironment of NSCLC patients after treatment. With this study, we hope to promote the application of neoadjuvant therapy of ensartinib for ALK-positive patients with advanced NSCLC and to promote the study of the characterization of the immune microenvironment of NSCLC patients after treatment with ALK inhibitors.

## 2 Case report

In this case, a 60-year-old female patient without a history of smoking underwent an enhanced computed tomography (CT) scan during a medical examination in August 2023, revealing a 20-mm-diameter mass in the left lower lung and enlarged mediastinal lymph nodes (stations 7 and 10). A lung puncture biopsy verified the diagnosis of lung adenocarcinoma. Furthermore, next-generation sequencing (NGS) of a 425-gene panel (ALK, EGFR, RET, MET, KRAS, etc.) was conducted to identify the patient’s mutated genes, microsatellite instability (MSI), and tumor mutational burden (TMB). An ALK fusion mutation involving EML4 exon 13 and ALK exon 20 was subsequently discovered. Since neoadjuvant therapy and surgical resection are recommended, a dose of 225 mg of ensartinib once daily was prescribed after the patient provided informed consent. The patient developed Grade 4 edema after three weeks of medication. The targeted therapy course was discontinued, and the patient was hospitalized for symptomatic treatment, including diuresis and dehydration. After one week, significant relief was observed, and ensartinib treatment was resumed.

**Figure 1:**
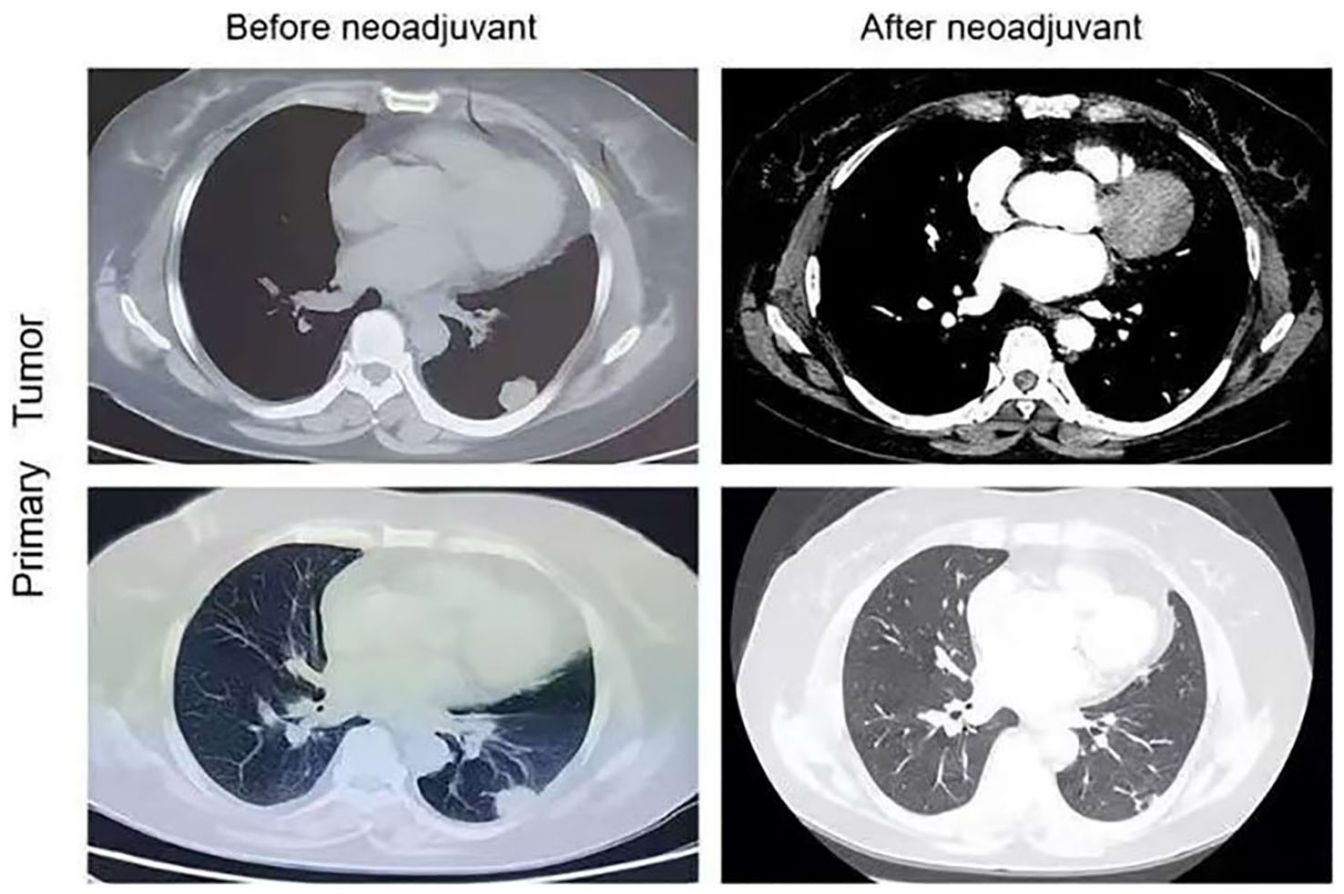
Chest CT images of left lung adenocarcinoma before and after the neoadjuvant therapy.

**Figure 2:**
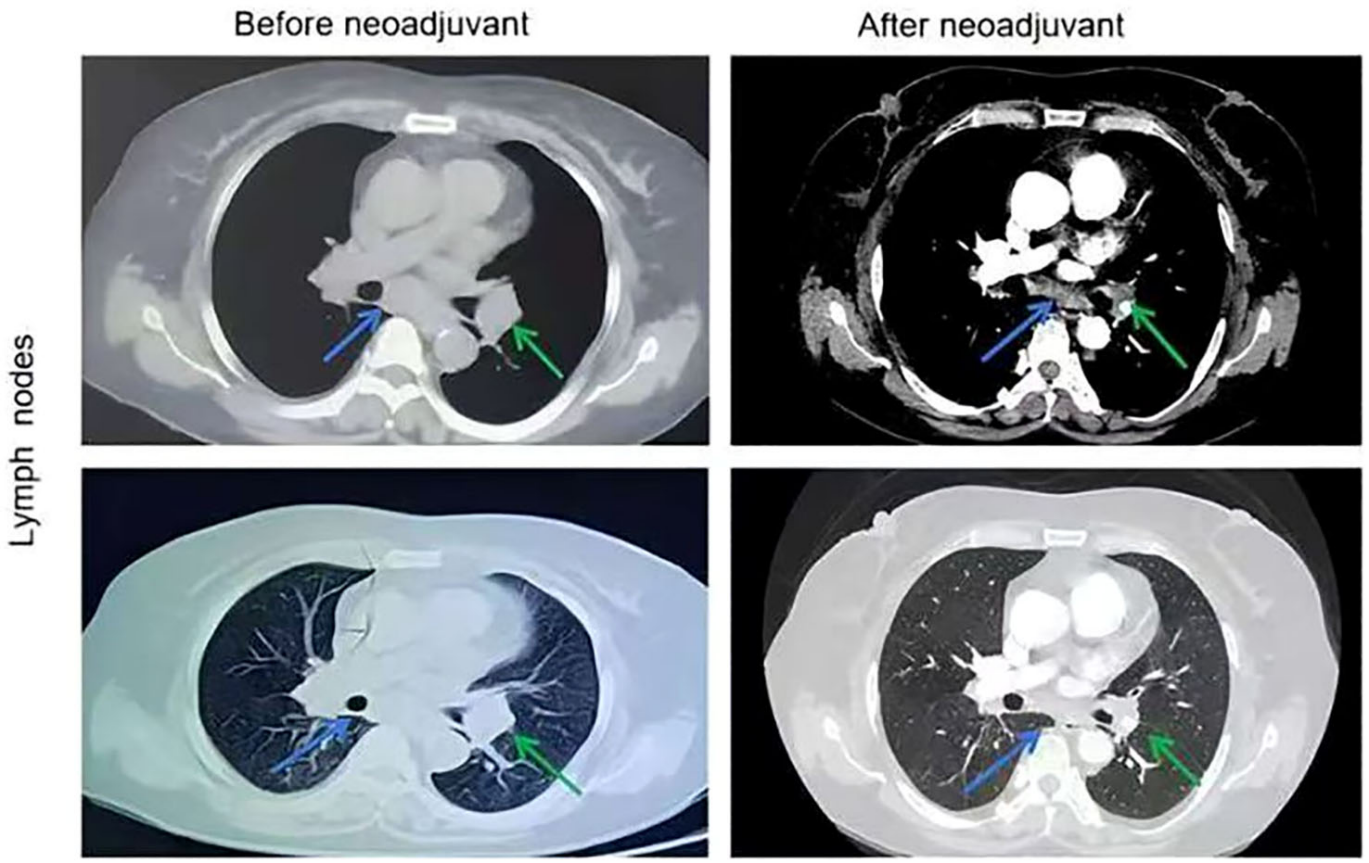
Chest CT images of lymph nodes before and after the neoadjuvant therapy. The stations 7 lymph nodes pointed by blue arrows and the stations 10 lymph nodes pointed by green arrows.

After three months of treatment, a chest CT scan revealed 50% tumor shrinkage and partial reduction of the mediastinal lymph nodes (**Figures 1**, **2**). After significant shrinkage of the tumor, the patient was proposed for surgery and was admitted to the hospital for preoperative investigations. Chest CT suggested that a soft tissue density nodular shadow was visible under the pleura of the lower lobe of the left lung, measuring about 14 mm×9 mm with multiple burrs at the margin.Tumor marker indices such as gastrin-releasing peptide precursor, cytokeratin 19 fragment, squamous cell carcinoma antigen and carcinoembryonic antigen were in the normal range.The patient subsequently underwent left lower lobectomy and systemic lymph node dissection without any serious perioperative complications. Final histopathological assessments revealed pT2aN2aM0 stage invasive adenocarcinoma. The tumor was solid, vesicular, and invaded the pleura, with visible spread through the air space; metastatic carcinoma was observed in Group 5 lymph nodes (1/3). No metastatic carcinoma was observed in groups 4, 7, and 9 or in the parabronchial lymph nodes. We performed a treatment response assessment in the postoperative pathological analysis, which suggested that the percentage of residual viable tumor cells was about 30% and the mesenchyme was about 70%, with no necrosis.Three days after the surgery, a follow-up chest CT showed normalization of the lungs, which was consistent with “postoperative left lung” changes.Postoperatively, the patient continued ensartinib therapy, and the dosage was changed to 125 mg once daily. Six months after surgery, the patient’s chest CT was repeated, suggesting an uneventful pulmonary recovery.Meanwhile,the patient has not experienced postoperative tumor progression or any severe drug-related adverse reactions.

**Figure 3:**
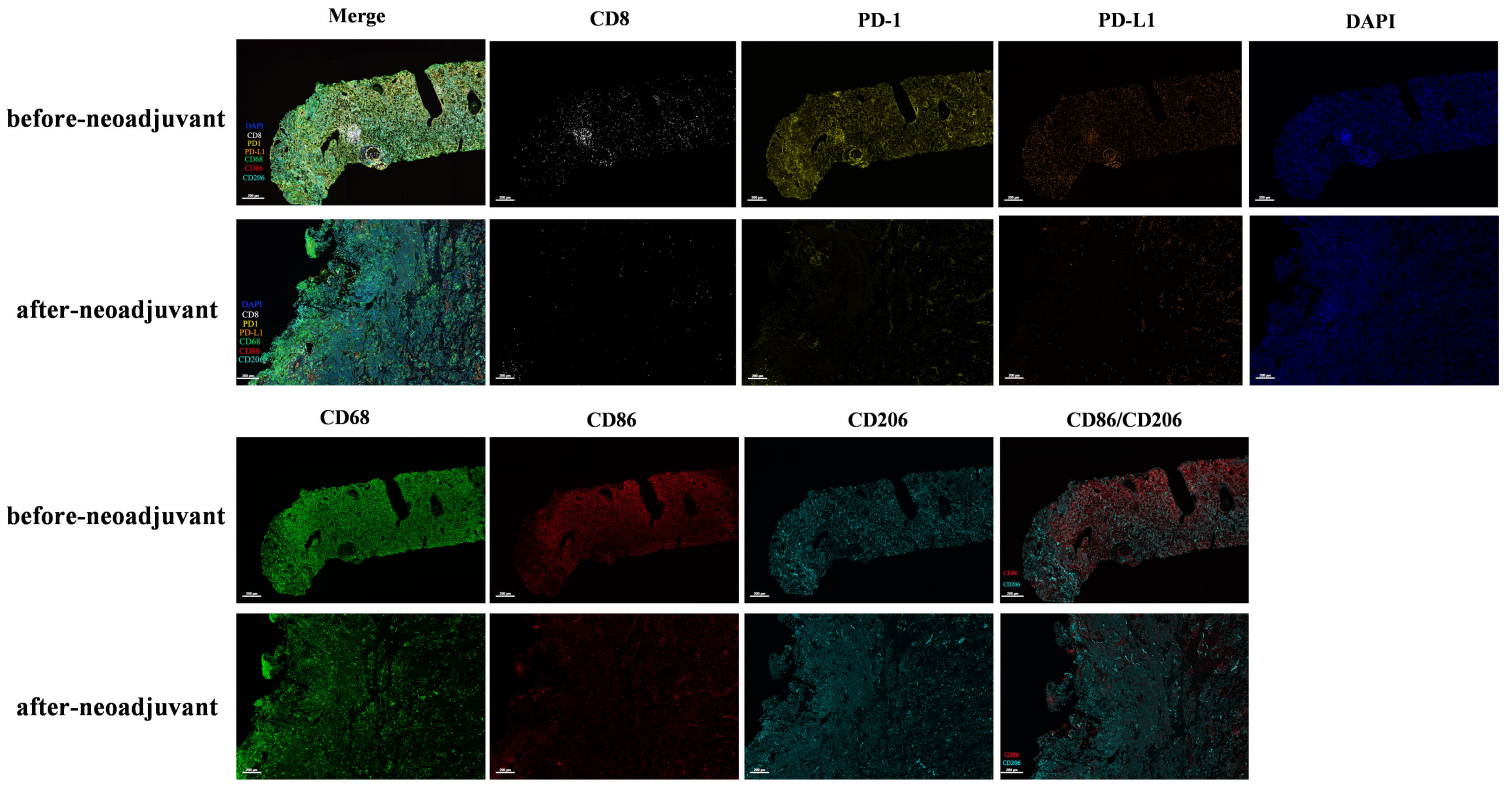
Multiplex immunofluorescence detection of immune microenvironment before and after neoadjuvant therapy.

**Figure 4:**
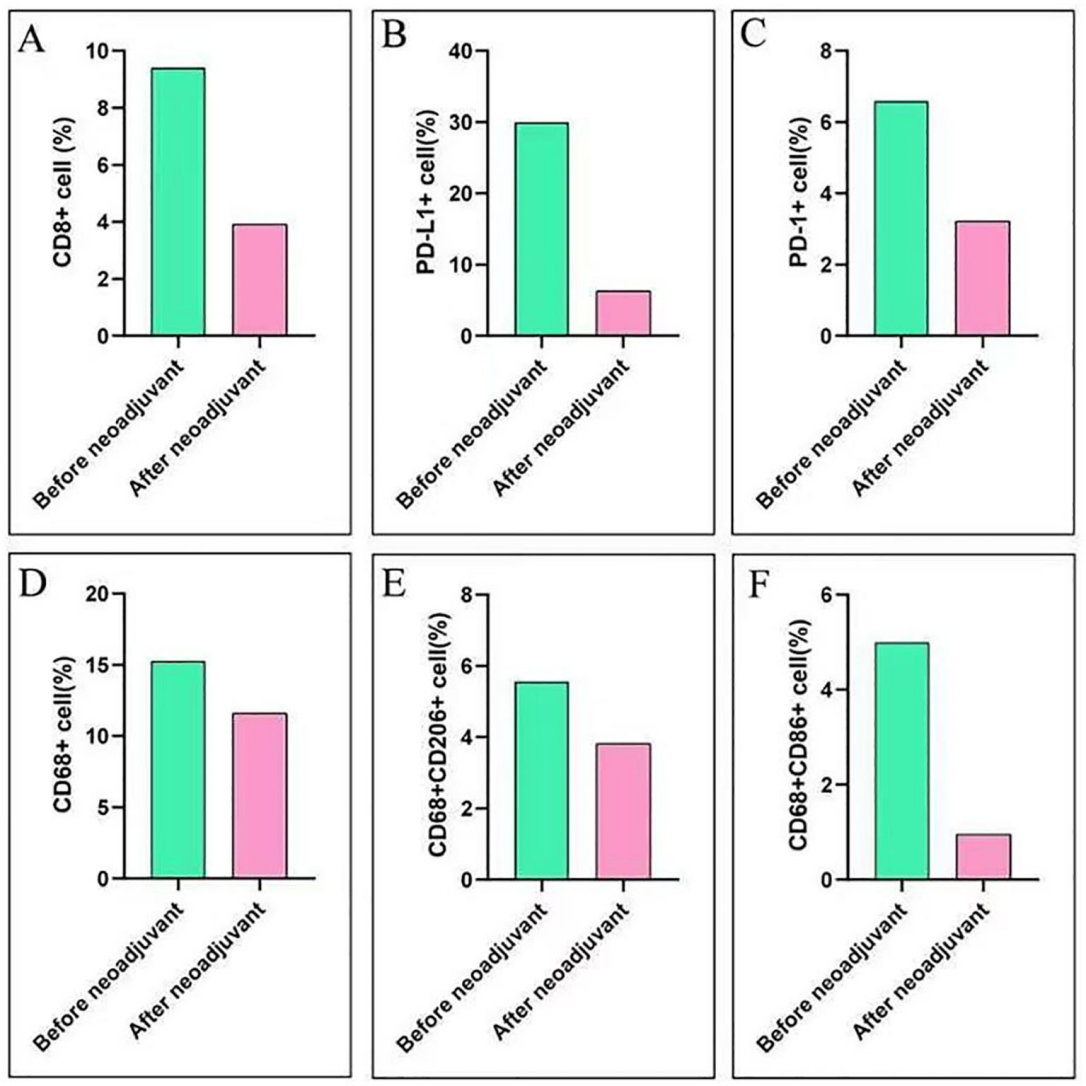
Immune score before and after neoadjuvant therapy. **(A)** Changes in CD8+ cell expression before and after neoadjuvant therapy; **(B)** Changes in PD-L1+ cell expression before and after neoadjuvant therapy; **(C)** Changes in PD-1+ cell expression before and after neoadjuvant therapy; **(D)** Changes in CD68+ cell expression before and after neoadjuvant therapy; **(E)** Changes in CD68+/CD206+ cell expression before and after neoadjuvant therapy; **(F)** Changes in CD68+/CD86+ cell expression before and after neoadjuvant therapy.

To investigate alterations in the tumor microenvironment (TME) before and after neoadjuvant ensartinib treatment, multiplex fluorescence staining was performed on pre-neoadjuvant and postoperative samples, with a focus on CD8+ T cells, macrophages, PD-1, and PD-L1 (**Figure 3**). We observed a significant reduction in the number of macrophages and CD8+ tumor-infiltrating lymphocytes (TILs) as well as PD-1 and PD-L1 expression. Additionally, the M1/M2 macrophage ratio also decreased (**Figure 4**).

## 3 Discussion

Neoadjuvant therapy can improve the survival rate of ALK-positive NSCLC patients. Previous studies have indicated that a majority of EML4-ALK fusion gene-positive NSCLC patients benefit significantly from molecularly targeted therapies (14–17). Herein, we report a case of ensartinib neoadjuvant treatment in an NSCLC patient with an EML4-ALK-positive mutation. Ensartinib demonstrated a significant therapeutic effect in this EML4-ALK fusion gene-positive NSCLC patient. The patient’s tumor significantly decreased after ensartinib treatment, followed by successful lobectomy and systemic lymph node dissection. Postoperative pathological examination revealed metastases in the group V lymph nodes (1/3). Moreover, the patient’s condition was relatively stable postoperatively, and regular follow-up was conducted.

The advent of precision medicine has improved insights into the treatment of NSCLC. The identification of driver genes and subsequent targeted therapies have expanded the treatment options for NSCLC patients (8). Nonetheless, in NSCLC patients with ALK rearrangements, single-agent ALK inhibitors are superior to chemotherapy in advanced or metastatic-stage patients (18). The clinical success of targeted therapies in advanced NSCLC patients has also played a role in their ongoing integration with neoadjuvant therapies. Several studies have suggested that neoadjuvant ALK-TKI targeted therapies are effective, thereby supporting the application of neoadjuvant ALK-directed therapies (19–21). However, the relevant guidelines have not yet advocated the use of ALK TKIs for the neoadjuvant treatment of early-stage ALK-positive NSCLC patients, and there is a lack of outcome data from large, randomized trials (22). There are two ongoing phase II trials of alectinib with neoadjuvant therapy for ALK-positive NSCLCs: the ALNEO (NCT05015010) and NAUTIKA1 (NCT04302025) trials. The ALNEO trial, aimed at examining potentially resectable stage III ALK-positive NSCLC patients, began in 2021 and is expected to be finished in 2026. The NAUTIKA-1 trial is being conducted for patients with resectable stage IB-IIIA ALK-positive NSCLC. It started in 2020 and is expected to be completed in 2029.

In a network meta-analysis, researchers included 12 RCTs involving 3169 patients with 8 treatment regimens, and compared the effects of multiple ALK inhibitors by overall survival (OS), progression-free survival (PFS), and objective remission rate (ORR). In terms of OS, alectinib ranked the highest, followed by ceritinib and ensartinib; in terms of PFS, ensartinib had a significant advantage and ranked the highest, followed by alectinib and brigatinib; in terms of ORR, alectinib ranked the highest, followed by ensartinib and lorlatinib (23). Thus, ensartinib has been evaluated in treatment-naïve ALK-positive NSCLC patients compared with other ALKIs, and has shown promising results as first-line systemic therapy, but the results remain to be confirmed by additional future studies.

None of the available clinical trials have assessed ensartinib as a neoadjuvant therapy for ALK-positive NSCLC patients. Although there are only two studies on ensartinib in combination with neoadjuvant therapy (24, 25), they did not mention the impact of ensartinib treatment on TME. Another previous study suggested that, compared with patients with EGFR/KRAS-positive NSCLC, patients with ALK-positive NSCLC have an immunosuppressive TME (26). However, large-scale cohort studies on how the TME of ALK-positive NSCLC changes after TKI treatment are lacking. Our study revealed that after neoadjuvant therapy, ALK-positive NSCLC transitions from an immunologically “hot” tumor to a “cold” tumor with decreased infiltration of CD8+ cells and macrophages, as well as a lower M1/M2 macrophage ratio (**Figures 3**, **4**). Pyo K-H et al. revealed that in ceritinib-resistant ALK-positive NSCLC, the CD8+ T-cell population remained unaffected (27). Kleczko EK et al. reported that T-cell infiltration in the TME of ALK-positive NSCLC remained unaffected, whereas macrophage infiltration decreased after alectinib treatment (28).

However, many studies have proposed contrasting findings. Fang Y et al. performed RNA sequencing on 8 patients with ALK mutations and obtained immune assessment scores, which revealed no difference in the T-cell and macrophage scores of the TME before and after neoadjuvant therapy (29). In a phase IB trial of alectinib plus atezolizumab in ALK-positive patients, CD8+ T cells were estimated before treatment, and after treatment with alectinib for seven days, CD8+ T-cell infiltration increased in seven of nine patients, but the T-cell increase was not associated with the therapeutic effect of anti-PD1 therapy (30). Similarly, Cao P et al. suggested that an ALK-positive patient on alectinib had an increased tumor inflammation signature score (31). In general, the changes in the immune microenvironment after neoadjuvant therapy in ALK-positive NSCLC patients remain ambiguous. This variability might be associated with tumor heterogeneity, treatment drugs and duration, and detection methods.

## 4 Conclusion

We found that ensartinib neoadjuvant targeted therapy is efficient in patients with EML4-ALK fusion gene-positive NSCLC. Additionally, surgical treatment after neoadjuvant therapy is safe and feasible. Neoadjuvant ensartinib treatment might be a better therapeutic intervention than conventional radiotherapy and chemotherapy, but achieving good therapeutic effects with ensartinib therapy combined with or sequential immunotherapy may be difficult. In the future, more clinical trials are needed to evaluate the effectiveness of ensartinib in neoadjuvant therapy and patients’ long-term prognosis, as well as to optimize treatment options.

## Data Availability

The original contributions presented in the study are included in the article/**Supplementary Material**. Further inquiries can be directed to the corresponding author.
